# Therapeutic Self-Disclosure within DBT, Schema Therapy, and CBASP: Opportunities and Challenges

**DOI:** 10.3389/fpsyg.2017.02073

**Published:** 2017-11-29

**Authors:** Stephan Köhler, Anne Guhn, Felix Betzler, Christian Stiglmayr, Eva-Lotta Brakemeier, Philipp Sterzer

**Affiliations:** ^1^Department of Psychiatry and Psychotherapy, Charité Universitätsmedizin Berlin, Berlin, Germany; ^2^Arbeitsgemeinschaft für Wissenschaftliche Psychotherapie, Berlin, Germany; ^3^Department of Clinical Psychology and Psychotherapy, Philipps University of Marburg, Marburg, Germany

**Keywords:** therapeutic self-disclosure, therapeutic alliance, CBT, third wave, DBT, schema therapy, CBASP

## Abstract

In recent years, various therapeutic interventions have been established that extended behavior and cognitive behavior therapy (CBT) by so-called “third-wave” strategies. In order to address specific therapeutic challenges in certain subgroups of patients who do not sufficiently respond to “classical CBT,” some of these third-wave strategies put particular emphasis on therapist self-disclosure. This article highlights therapeutic self-disclosure as a means to address interpersonal problems by comparing three third-wave strategies: (a) acceptance and change strategies as used in Dialectical Behavioral Therapy (DBT), (b) the concept of “limited reparenting” as used in Schema Therapy (ST), and (c) disciplined personal involvement as used in the Cognitive Behavioral Analysis System of Psychotherapy (CBASP). On the basis of a critical discussion on opportunities and challenges within these three concepts, self-disclosure is proposed to be a promising therapeutic tool that is worth to be investigated in more depth in future studies.

## Background

Despite the1pc2pc indisputable success of cognitive behavior therapy (CBT) in the treatment of many psychiatric disorders, its effectiveness turned out to be limited in some patient groups, e.g., patients with borderline personality disorder (BPD) or chronic depression (CD; Kriston et al., 2014). These limitations gave rise to the “third-wave” of CBT with the development of new therapeutic strategies such as Dialectical Behavioral Therapy (DBT; Linehan, 1993) or Schema Therapy (ST; Young, 1994) for BPD and Cognitive Behavioral Analysis System of Psychotherapy (CBASP; McCullough, 2000) for CD. These third-wave approaches may be viewed as enhanced versions of CBT that focus more strongly on emotions and their regulation as well as the therapeutic relationship (Kahl et al., 2012). One key aspect of the therapeutic relationship that became particularly important in this regard is self-disclosure by the therapist.

In general, therapist self-disclosure simply refers to all statements that reveal something personal about the therapist (Hill and Knox, 2002). It can serve a variety of purposes (see Knox and Hill, 2003) such as the strengthening of mutual trust or the validation of the patients’ reactions, e.g., by disclosing one’s own feelings. Although self-disclosure is used to some degree in 90% of psychotherapies (Edwards and Murdock, 1994), specific aspects of self-disclosure (form, frequency, aim, point of time) against the theoretical background for its application have been addressed far too little (Henretty and Levitt, 2010). Two randomized controlled trials (RCTs) investigated the effectiveness of therapist self-disclosure empirically and found that patients receiving psychotherapy with heightened therapist self-disclosure report lower levels of symptom distress and more affection for the therapist than patients in restricted disclosure conditions (Barrett and Berman, 2001; Ziv-Beiman and Shahar, 2016). From the patients’ perspective, self-disclosures make therapists seem more real and human, which in turn strengthens the therapeutic alliance (Knox et al., 1997). The present paper, however, aims at a more precise definition of self-disclosure that is providing feedback on the interpersonal impact of the patient’s behavior on the therapist. Regarding the different subtypes of self-disclosure established by Knox and Hill (2003), this definition refers to ‘self-disclosure of immediacy.’ It has been utilized to build a methodical technique in certain third-wave CBT, namely DBT, ST, and CBASP. Especially, in the treatment of patients with personality disorders in which interpersonal problems are a defining feature, this form of therapist self-disclosure is considered a key element since the patient-therapist-interaction most likely parallel the interpersonal problems with others in the patient’s life (Knox and Hill, 2003). Going beyond the more general purpose of strengthening the therapeutic alliance, this type of self-disclosure specifically relates to therapist-patient interaction. This is in some contrast to mindfulness focussed third-wave strategies such as Acceptance and Commitment Therapy (ACT, Hayes et al., 1999) or Metacognitive Therapy (MCT, Wells and Matthews, 1994). Here, therapeutic alliance is of a high relevance, too (Vilardaga and Hayes, 2009). It provides the basis for helping patients to overcome negative thoughts and feelings by using strategies such as metaphors, paradoxes, and experiential exercises. However, these strategies are not intended to focus on the patient-therapist interaction itself. In the following, we use DBT, ST, and CBASP as three examples to illustrate the concept of therapeutic self-disclosure as a therapeutic instrument. Of note, the focus on these three third-wave therapies is not at all meant to be exhaustive. There are other treatments that also use self-disclosure as a therapeutic instrument to some degree, but we here focus on those psychotherapies that in our view most directly and explicitly describe and use self-disclosure to address the patient’s interpersonal problems. However, no direct empirical evidence is currently available for the effectiveness of self-disclosure within these strategies. But, as self-disclosure seems to be a relevant factor for the quality of the therapeutic alliance within these strategies (Knox et al., 1997; Knox and Hill, 2003), we will briefly review studies that examined the therapeutic alliance and may therefore hint at a relevant role of self-disclosure. Finally, we discuss opportunities and challenges in the use of therapeutic self-disclosure, and highlight questions that will need to be addressed by future research.

## Self-Disclosure in Dialectical Behavioral Therapy (DBT)

Dialectical Behavioral Therapy was originally developed as a specific strategy to address the problem of frequent disruptions in the therapeutic relationship with BPD patients in classical CBT (Linehan, 1993). An essential element of DBT is the therapeutic alliance (Linehan, 1993; Barnicot et al., 2012). The basic idea is related to the classic definition of dialectic, which comprises two aspects: acceptance and change. In addition to the dialectical view of life, dialectic is implemented in the therapist-patient-communication and in the formation of a therapeutic alliance. The therapeutic relationship is thus viewed dialectically, defined as a permanent and polarizing interplay between *acceptance* and *change* by communicating to the patient that the therapist accepts the way she/he is, while at the same time pushing her/him for change. Therefore, DBT offers strategies that focus either on acceptance or on change. Self-disclosure is an important aspect in the therapeutic alliance of DBT. It can also be separated in disclosure of personal information about the therapist (e.g., past experiences, professional activities, family status) and disclosure of the therapist’s immediate, personal reactions to the patient within the therapeutic interaction. As we focus on self-disclosure as a therapeutic tool to focus on interpersonal problems, we highlight the corresponding DBT elements. *Acceptance strategies* include validation techniques, flexible therapeutic offers (e.g., phone calls), and importantly, self-disclosure by the therapist (e.g., “I really like working with you”). *Change strategies* comprise, besides the use of skills and behavioral analyses, a confrontational communication style that aims at modulating a (grid-locked) affect and at taking a different perspective, which also includes self-disclosure by the therapist (“If you are calling me every day I may get upset about your phone calls – which I would regret a lot because I would like to keep supporting you with phone calls.”). Self-disclosure is thus an important part in this dialectic therapist-patient-communication and can support either acceptance or change. An inherent risk of this approach is a potential imbalance between acceptance and change, which might in turn aggravate the patient’s emotional dysregulation. In the framework of the dialectic approach it is also necessary to use self-disclosure by emphasizing one’s own therapeutic boundaries, e.g., by disclosure of personal weaknesses.

The effectiveness of DBT has been proven by several RCTs and meta-analyses (Linehan et al., 2006; Stoffers et al., 2012). However, little is known about the contribution of self-disclosure to these effects. In an RCT, patients’ ratings of therapist commitment and working capacity were associated with fewer suicide attempts and commitment was associated with reduced non-suicidal self-injury, thus pointing to a relevant effect of the therapeutic alliance (Bedics et al., 2015). Similarly, a naturalistic study showed that DBT high-responders identified an understanding, empathic and accepting alliance between patient and therapist as supporting factors in the therapeutic process (Stiglmayr et al., 2014). While these results emphasize the importance of a strong therapeutic alliance in the treatment of patients with BPD, the effect of self-disclosure in DBT remains to be investigated.

## Self-Disclosure in Schema Therapy (ST)

The original ST concept was principally established by Young et al. (2003) and aimed at identifying maladaptive schemas. An important concept in ST is the “schema mode model.” A schema mode represents “schemas, coping responses, or healthy behaviors that are currently active for an individual.” A focus in ST is emotion-focused work that aims to strengthen the patients’ personality structure, in particular the integration of the different modes under the control of the “healthy adult.” Therapist self-disclosure is an essential means to achieve these goals. In this regard, “limited reparenting” plays a special role (Alexander and French, 1946). Here, frustrated basic needs in the patient’s childhood should be met within the therapeutic relationship while at the same time dysfunctional behaviors should be addressed through empathic confrontation.

Self-disclosure in ST focuses on early maladaptive schemas. For example, in the domain “mistrust/abuse,” the therapist explicitly behaves in a trustworthy and sincere way, addressing any negative feelings or expectations of the patient toward the therapist. In the domain “inadequacy and shame,” reparenting means that the therapist demonstrates acceptance and a non-judgmental attitude. In the framework of limited reparenting, self-disclosure by the therapist plays a key role. Depending on the patient’s specific early maladaptive schema, the therapist may disclose own emotions, thoughts, and experiences, e.g., “I did not have much affection either as a child. That’s why I know how important it is to get that affection” (Young et al., 2003). During the course of treatment, patients internalize the therapist’s behavior as part of their own healthy adult mode (Young et al., 2003). Support and setting limits have to be balanced in a flexible way. Too much reparenting may impede the formation of a healthy adult mode, thus blocking the therapeutic process (Roediger and Zarbock, 2015). Overly restrictive boundaries may lead to excessive demands on the patient’s side, causing feelings of guilt in the therapist.

Evidence for the efficacy of ST was found in two RCTs conducted in BPD patients (Giesen-Bloo et al., 2006; Farrell et al., 2009) and in an RCT for six other personality disorders (Bamelis et al., 2014). While the specific role of self-disclosure in the effectiveness of ST has not been studied to date, evidence indicates that the therapeutic alliance in general is particularly strong in ST and predicts clinical improvement (Spinhoven et al., 2007).

## Self-Disclosure in the Cognitive Behavioral Analysis System of Psychotherapy (CBASP)

Cognitive behavioral analysis system of psychotherapy was specifically developed for the treatment of CD patients by McCullough (2000). It integrates cognitive-emotional, behavioral, interpersonal, and psychodynamic theories and treatment strategies to address the specific psychopathology of CD. The interpersonal behavior of CD patients is typically characterized by prelogical and precausal thinking, resistance to logical reasoning or reality-based feedback, egocentricity, talking in monolog, lack of empathy, and emotional dysregulation under stress (McCullough, 2000; Kuhnen et al., 2011). These behaviors are often a source of frustration in relationships with others, including the therapist. McCullough (2000) proposes that the therapeutic relationship is instrumental in re-connecting patients with their environment and in promoting healing interpersonal experiences against the background of early traumata. As opposed to the neutral attitude of therapists in classical CBT, CBASP therapists behave in a more personal way by providing self-disclosure in the form of so-called ‘disciplined personal involvement’ (DPI: McCullough, 2006). Classical CBT does not exclude the possibility to use therapist self-disclosure to model effective coping, however, CBASP explicitly presupposes therapists’ personal involvement as key element in treating CD patients. In response to interpersonal problems in the therapeutic setting, the therapist directs the focus of attention to the patient-therapist dyad [for example: “You refuse to make eye contact with me. You make me feel that I don’t exist.” (McCullough et al., 2015)]. Self-disclosure requires therapists to continuously adjust their interpersonal style by considering their patients’ learning history with the so-called ‘significant others’ (e.g., their parents). The impact of DPI derives from the therapist’s non-complementary behavior in these hot-spot situations. To contrast the therapist’s actual response with the response expected on the basis of earlier experiences with significant others, a second technique is applied, the ‘interpersonal discrimination exercise’ (IDE). Therapists teach their patients to recognize corrective cognitive-emotional and behavioral responses that allow them to participate in the interpersonal reality (for example: “What has been my response to your telling me about what happened? What is different about my reaction to you and the reactions you got from your mother?,” McCullough et al., 2015). DPI and IDE are applied multiple times throughout therapy. Therapists are required to be themselves with patients in the way that they respond naturally and in an explicitly non-neutral but self-disciplined manner (McCullough, 2006). In order to identify personal hot-spots and to preclude undisciplined countertransference, CBASP therapists must have a high degree of self-awareness in terms of their individual interpersonal learning history.

Although several RCTs and open studies (Kocsis et al., 2009; Schramm et al., 2011, 2017; Wiersma et al., 2014; Brakemeier et al., 2015) have demonstrated effectiveness of CBASP, the precise active factors are still a matter of debate. In particular, no empirical data are currently available to support a specific effect of therapeutic self-disclosure, despite its central role in CBASP. However, there is some empirical evidence that underlines the importance of the therapeutic alliance in CBASP. The quality of the therapeutic alliance early in the course of CBASP seems to predict treatment outcome independent of other outcome-relevant factors (Klein and Santiago, 2003; Arnow et al., 2013). A stronger early therapeutic alliance predicts decrease in patients’ hostile and submissive interpersonal styles, which are in turn related to depressive symptom improvement at the end of treatment (Constantino et al., 2016). In light of the idea that self-disclosure is an important factor in enhancing the therapeutic alliance (Knox and Hill, 2003), it is tempting to speculate that self-disclosure may contribute to these effects. While this question still awaits direct empirical investigation, it is interesting to note in this regard that higher reactance levels in terms of the unwillingness to receive input by others as well as oppositional tendencies positively predict treatment outcome in CBASP (Arnow et al., 2003). This suggests that self-disclosure techniques such as DPI and IDE, which are integral parts of CBASP, may be beneficial in that they help the therapist to handle perturbations in the therapeutic relationship in a constructive way.

## Comparing Therapeutic Self-Disclosure in DBT, ST, and CBASP – Opportunities and Challenges

Maladaptive interpersonal styles are typically evident in personality disorders, particularly BPD, or CD and manifest themselves in psychotherapy. In contrast to the first and second-waves of CBT, certain third-wave strategies (DBT, ST, and CBASP) propose therapist self-disclosure as a key instrument to address interpersonal problems. These strategies share the importance of therapeutic self-disclosure and equip the therapist with specific techniques to address maladaptive interpersonal styles that can hinder the therapeutic process. However, based on the patients’ specific characteristics and needs that these strategies target, there are also significant differences between DBT, ST, and CBASP in their use of therapeutic self-disclosure. In ST, the concept of “limited reparenting” allows self-disclosure primarily in terms of disclosing positive emotions of acceptance and warmth to overcome frustrated basic needs of the patients’ child mode. Self-disclosure in ST is further intended to provide a role model until patients can build a “healthy adult mode” themselves. In DBT, acceptance and change strategies utilize self-disclosure to cope with auto-aggressive as well as destructive behavior that hampers the therapeutic process. Self-disclosure in DBT comprises thus both, positive as well as negative emotions. Self-disclosure in CBASP goes somewhat beyond its use in DBT and ST. It is aimed at hot-spot situations in which patients expect the therapist to behave in a way that resembles the behavior of significant others during the patients’ development. The disclosure of positive and negative feelings is intended to make interpersonal consequences transparent. In CBASP, self-disclosure is viewed as an essential precondition to reconnect CD patients with their interpersonal environment and thus creating healing relationship experiences against the background of early traumata.

All three third-wave strategies discussed here are oriented toward the adaptive regulation of emotions that are thought to underlie maladaptive interpersonal behavior. The dialectic in DBT allows to compensate strong oscillations between attachment and detachment in patients with BPD and sustains the process between acceptance and change. The strategy of “limited reparenting” in ST allows to process difficult emotional states that comprise functional or dysfunctional modes and that may be encouraged or restrained, respectively. In CBASP, emotional activation in hot-spot situations is addressed by self-disclosure (DPI) and by discrimination (IDE) between the significant-others behavior and the therapist’s responses to explicitly practice emotion regulation.

To implement these forms of self-disclosure, several factors are necessary: biographical work, especially with focus on childhood experiences and interpersonal learning history, plays a considerably greater role in these treatment strategies than in classical CBT. Without reference to childhood experiences, the specific use of self-disclosure as a tool to enable healing interpersonal experiences would not be possible. Furthermore, the therapist’s willingness to engage in self-disclosure is needed. A high degree of self-awareness and regular supervision are indispensable. A risk of focusing on self-disclosure might be that other important and effective techniques of CBT such as behavioral activation, cognitive restructuring, or exposure therapy may be neglected. Moreover, self-disclosure is an emotional challenge for therapists and poses the risk of overemphasis on the therapist’s feelings. Another aspect is the risk that crises in the therapeutic relationship can be triggered by the use of self-disclosure. However, there is some evidence that the occurrence of “positive” and “negative” phases in the therapy is associated with a favorable treatment outcome (Safran et al., 2011).

## Concluding Remarks

In our opinion, therapeutic self-disclosure as a key component of certain third-wave CBT strategies is a promising therapeutic tool for the treatment of patients who suffer from chronic and disabling disorders with high rates of treatment resistance. Within third-wave CBT, different strategies have developed different ways of using self-disclosure as a therapeutic instrument. However, the question arises whether self-disclosure is a “conditio sine qua non” in modern psychotherapy. A recent Cochrane Review (Olthuis et al., 2015) examined the results of several studies that compared therapist-supported internet-treatment (ICBT) with classic “face-to-face” CBT in patients with anxiety disorders. Overall, ICBT was found to be effective (compared to waiting list) in this meta-analysis, but no significant difference between ICBT and classical CBT was detected. A key factor in determining the usefulness of self-disclosure could be whether a disorder is characterized by maladaptive interpersonal behavior. It should be noted, however, that this assertion must currently remain speculative, as evidence from RCTs is lacking to date. Further research is needed to evaluate the efficacy of self-disclosure, e.g., with so called dismantling studies (Høglend, 2014). A feasible design regarding the psychotherapeutic concepts reviewed here would include the comparison of, for example, CBASP with and without DPI, to evaluate the impact and possible side effects of therapeutic self-disclosure. Further aspects that should be investigated include the precise form and frequency of self-disclosure and the effect of these factors on treatment outcome.

## Author Contributions

SK conceived the contents and structure of the article. SK, AG, and PS co-wrote the paper. FB edited the introduction, CS edited the DBT part and E-LB edited the whole manuscript. Critical revision of the manuscript for important intellectual content: SK, AG, FB, CS, E-LB, and PS.

## Conflict of Interest Statement

The authors declare that the research was conducted in the absence of any commercial or financial relationships that could be construed as a potential conflict of interest.
